# Environmental Pollutants as Emerging Concerns for Cardiac Diseases: A Review on Their Impacts on Cardiac Health

**DOI:** 10.3390/biomedicines13010241

**Published:** 2025-01-20

**Authors:** Vinay Kumar, Hemavathy S, Lohith Kumar Dasarahally Huligowda, Mridul Umesh, Pritha Chakraborty, Basheer Thazeem, Anand Prakash Singh

**Affiliations:** 1 Biomaterials & Tissue Engineering (BITE) Laboratory, Department of Community Medicine, Saveetha Medical College and Hospital, Saveetha Institute of Medical and Technical Sciences (SIMATS), Thandalam, Chennai 602105, Tamil Nadu, India; vinaykumar.smc@saveetha.com (V.K.);; 2 Institute for Water and Wastewater Technology, Durban University of Technology, Durban 4001, South Africa; 3 Department of Life Sciences, Christ University, Hosur Road, Bengaluru 560029, Karnataka, India; 4 Area of Molecular Medicine, Department of Allied Healthcare and Sciences, JAIN (Deemed to be University), Bangalore 560066, Karnataka, India; 5 Waste Management Division, Integrated Rural Technology Centre (IRTC), Palakkad 678592, Kerala, India; 6 Frankel Cardiovascular Center, Department of Medicine, University of Michigan, Ann Arbor, MI 48109, USA

**Keywords:** cardiovascular diseases (CVDs), environmental pollutants, cardiac health, health risk factors, particulate matter

## Abstract

Comorbidities related to cardiovascular disease (CVD) and environmental pollution have emerged as serious concerns. The exposome concept underscores the cumulative impact of environmental factors, including climate change, air pollution, chemicals like PFAS, and heavy metals, on cardiovascular health. Chronic exposure to these pollutants contributes to inflammation, oxidative stress, and endothelial dysfunction, further exacerbating the global burden of CVDs. Specifically, carbon monoxide (CO), ozone, particulate matter (PM_2.5_), nitrogen dioxide (NO_2_), sulfur dioxide (SO_2_), heavy metals, pesticides, and micro- and nanoplastics have been implicated in cardiovascular morbidity and mortality through various mechanisms. PM_2.5_ exposure leads to inflammation and metabolic disruptions. Ozone and CO exposure induce oxidative stress and vascular dysfunction. NO_2_ exposure contributes to cardiac remodeling and acute cardiovascular events, and sulfur dioxide and heavy metals exacerbate oxidative stress and cellular damage. Pesticides and microplastics pose emerging risks linked to inflammation and cardiovascular tissue damage. Monitoring and risk assessment play a crucial role in identifying vulnerable populations and assessing pollutant impacts, considering factors like age, gender, socioeconomic status, and lifestyle disorders. This review explores the impact of cardiovascular disease, discussing risk-assessment methods, intervention strategies, and the challenges clinicians face in addressing pollutant-induced cardiovascular diseases. It calls for stronger regulatory policies, public health interventions, and green urban planning.

## 1. Introduction

Cardiovascular diseases include heart failure, hypertension, arrythmia, chronic coronary artery diseases, and stroke, which are classified as noncommunicable diseases. The World Health Organization (WHO) has estimated that 80% of worldwide annual deaths will be due to noncommunicable diseases in 2030 [1]. The highest number of deaths is caused by cardiovascular diseases within noncommunicable diseases. Data provided by Global Burden of Diseases (GBD) showed a 30% increase in cardiovascular diseases in 2019 compared to 1990, and this is expected to increase the burden of global economic costs by USD 47 trillion within the next 20 years [2]. Environmental pollution is one of the major but most ignored causes of cardiovascular diseases, as compared to diseases in the general population. It is time to prioritize investigations of the long-term health effects of environmental pollution through a multi-exposure perspective named the exposome concept [3]. The exposome of an individual is characterized by external environmental risks, lifestyle, socioeconomic situation as a whole. The exposome concept was created to address the multi-exposure conditions defined by domains of natural, personal and social environments on health [4].

**Figure 1:**
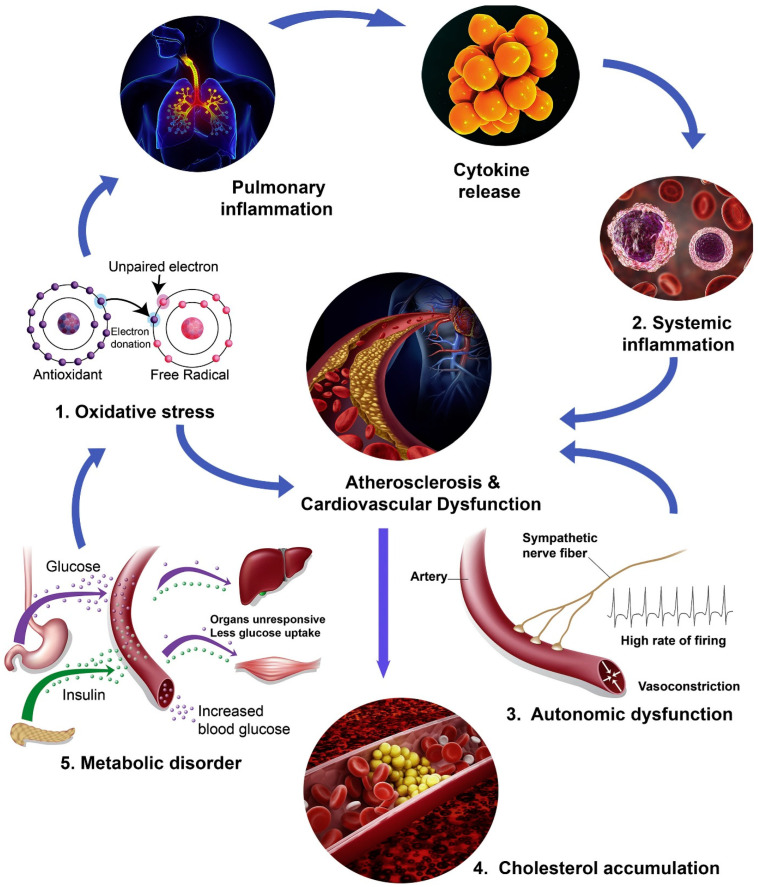
Effect of exposure of particulate matter and associated cardiovascular disease. Reprinted with permission [9].

Industrialization and urbanization have generated anthropogenic air pollutants that change the nature and composition of air pollution, projecting it as a major public health concern. The components of air pollutants arise from complex chemical reactions and cannot be classified on the basis of mass, size or surface chemistry. Air pollutants can be generally gaseous pollutants (ozone, nitrogen oxide, sulfur oxide and carbon monoxide) [3]. Chronic air-pollutant exposure affects life expectancy and increase the chances of CVD. Particulate matter is a mixture of different-sized particles with different compositions, sources and biological effects. They can typically be characterized as organic products from fuel combustion, water, inorganic matter and heavy metals [5]. Particulate matter with a 0.1 µm diameter is termed ultrafine particles and has more particle numbers, an increased surface-to-mass ratio, a reactive surface and high solubility. These characteristics contributes to higher potency towards toxic cardiovascular capacity by increasing alveolar penetration, organ damage and systemic circulation. Particulate matter with a 2.5 µm diameter (PM_2.5_) is generated from fuel combustion, vehicle exhausts and industrial processes [6]. A study by [7] showed that PM_2.5_ exposure increases the risk of hypertension, arrythmias, endothelial dysfunction, coagulation, thrombosis and atherosclerosis. Short-term exposure can trigger plaque rupture and cause myocardial infarction, while long-term exposure leads to atherosclerosis. Recent increases in wildfires around the world have increased the release of PM_2.5_ in the air, affecting countries like China and India. According to a report [8], it has been observed that the cases of wildfires have increased due to generated PM_2.5_. The composition of PM_2.5_ generated from wildfires differs from the ambient air and contains gaseous pollutants, which are products from the incomplete combustion of sugar, lignin, inorganic salts, resins and waxes. Wildfire PM_2.5_ exposure is linked with myocardial infarction, cardiac arrest, stroke, and hypertension. The increased occurrences of wildfires are also emerging as threats to cardiac health. The exposure to and associated effects of PM_2.5_ in relation to cardiovascular disease are presented in Figure 1.

Climate change and changing temperature have a direct impact on cardiovascular health. Climate change causes frequent floods, hurricanes, droughts, extreme weather conditions, and the presence of mold and pollens in the air, which affect human cardiovascular health. However, extreme temperature has been identified as one of the most challenging and direct effects of climate change [10]. A recent study by [11] showed that an increase in temperature of 1 °C is associated with 0.5% increase in cardiac arrests and related diseases. A study conducted across 567 cities around 27 countries showed a 99% correlation between increases in temperature and humidity and increased occurrences of ischemic heart diseases, stroke and heart failure [12]. Extreme cold weather is linked with CVD-related hospitalization, risk of death and mortality. High temperature exposure increases core body temperature, dehydration, endothelial cell activation, heart rate and sympathetic activation as compared to extreme cold temperature, which leads to vasoconstriction, high blood pressure and electrophysiological disturbances [13]. Older adults (over 65 years) are most affected by the extreme change in temperature. Older individuals with pre-existing risk factors like diabetes, obesity, and hypertension have a higher risk of cardiovascular disease [10].

Other widely used chemicals named per- and polyfluoroalkyl substances (PFASs) are absorbed through oral, nasal and dermal routes and are deposited in the kidney, blood and liver, but they are not metabolized [14]. Humans are exposed to PFASs through water, food, household dust, indoor and ambient air, and PFAS-containing materials. Newer versions of PFASs are used widely and found in higher concentrations in serum, as the health risks associated are not clear [15]. The increased presence of PFASs in serum triggers hyperlipidaemia and activates platelets and coagulation along with oxidative damage through elevated levels of reactive oxygen species and endoplasmic reticulum stress [16]. PFASs are reported to increase endothelial permeability, which is a known driver for atherosclerosis, stroke and coronary heart diseases. PFASs are responsible for direct vasculo-toxicity and inflammatory effects, which are directly linked to CVD risk factors and occurrences of CVD [17].

The American Heart Association has included heavy metals as CVD risk factors. Humans are exposed to metals through ingestion and air pollution. Particulate matter carries lead, cadmium, arsenic, iron, zinc and nickel [18]. These metals can replace essential elements and create oxidative stress by forming reactive oxygen species. Oxidative stress can lead to lipid peroxidation, oxidative stress, disturbed lipid metabolism and elevated endothelial permeability, which are all associated with CVD risks [19]. The presence of heavy metals in blood, even in low concentrations, is connected to hypertension and obesity. The presence of lead and mercury is associated with increased levels of lipids in blood, while cadmium is related to increased levels of c-reactive protein, total cholesterol, and white blood cells, leading to atherosclerosis. Heavy-metal presence in lower concentrations than safety standards is related to coronary and peripheral artery diseases, as well as hypertension [20].

Increased cardiovascular mortality has been associated with noise pollution in road traffic [21]. Chronic stimulations of the stress hormones promote hypertension, hyperglycaemia and hyperlipidaemia—known cardiovascular disease risk factors [22]. Studies on rodents showed that chronic noise exposure causes hypertension endothelium-dependent relaxation and vascular inflammation due to reactive oxygen species, leading to atherosclerosis [23]. Sleep disturbance is another key factor in impaired cardiovascular health. Sleep disturbance and fragmentation are associated with haematopoiesis and circulating monocytes developing atherosclerotic lesions [24].

The exposome of an individual is related to various physical and environmental exposures, unhealthy city designs, industrialization, climate change and physiological and pathophysiological changes due to lifelong exposure to environmental stressors. This is something that can be assessed [25]. Exposure to multiple factors like air pollution, light pollution and noise pollution activate pathological mechanisms. This triggers the risk factors of CVDs, including oxidative stress, an increased supply of immune cells and vascular endothelial dysfunction [26]. Studies have shown that multi-hit environmental stressors are more common, but the totality of environmental stressors cannot be evaluated throughout the life [27].

## 2. Major Classes of Environmental Pollutants and Their Role in Cardiac Diseases

### 2.1. Particulate Matter

Particulate matter with a diameter of ≤2.5 µm (PM_2.5_) is a major contributor to air pollution, originating from industries, vehicles and wildfire smoke. PM_2.5_ poses significant health risks, particularly to the cardiovascular and pulmonary systems. Even at low exposure levels, PM_2.5_ is associated with increased morbidity and mortality rates.

A systematic review demonstrated a global association between heart failure and air pollution, and increased PM_2.5_ levels lead to an increased risk of CVD mortality [28]. Epidemiological studies consistently support these findings, emphasizing that even PM_2.5_ levels below the WHO guideline of 10 μg/m^3^ can contribute to cardiovascular issues. For instance, higher coronary calcium scores have been observed at exposures around 6.9 μg/m^3^, suggesting there may be no safe threshold for PM_2.5_ exposure regarding cardiovascular health [28,29]. PM_2.5_ exposure triggers the generation of reactive oxygen species (ROS), leading to a cascade of events including oxidative stress, inflammation, endoplasmic reticulum stress, and autophagy. These processes contribute to vascular calcification and atherosclerosis [29]. The toxicity of PM_2.5_ depends on particle size and composition. Hazard ratios for CVD, coronary heart disease, and cerebrovascular disease hospitalizations ranged from 1.04 to 1.07 per 4.0 μg/m^3^ PM_2.5_ increase. Analyses using residential-level PM_2.5_ and individual-level confounder data showed stronger associations compared to those using ZIP code-level data, indicating that precise exposure assessments are crucial [30]. Higher levels of PM_1_, PM_2.5_, and PM_10_ have been linked to increased risks of both metabolic syndrome (MetS) and CVD. Odds ratios ranged from 1.20 to 1.36 for MetS and hazard ratios ranged from 1.18 to 1.28 for CVD. Importantly, MetS was found to mediate the relationship between PM exposure and CVD, with mediation proportions of 3.7% for PM_1_ and 2.5% for PM_2.5_ in single mediator models. This suggests a comprehensive pathway: PM exposure triggers oxidative stress and inflammation, leading to the development of MetS, characterized by hypertension, dyslipidaemia, obesity, and insulin resistance, which in turn increases CVD risk. Over 10% of PM–CVD associations are associated with metabolic disorders, including obesity and blood pressure [31]. In an animal model, PM_2.5_ exposure in coal-combustion areas during the heating season resulted in significant cardiac effects, including impaired systolic function, myocardial fibrosis, and left ventricular enlargement. The inflammatory chemokine Ccl2 played a crucial role in mediating myocardial fibrosis by recruiting inflammatory cells and promoting collagen deposition. A study demonstrated identification of 171 suspect compounds in PM_2.5_ samples that had 40 active components [32]. A relationship was investigated between PM_2.5_ chemical components and cardiovascular hospitalizations across 184 Chinese cities. It focused on six specific components: black carbon (BC), sulfate (SO_4_^2−^), ammonium (NH_4_^+^), nitrate (NO_3_^−^), organic matter (OM), and chloride (Cl^−^). Significant associations were found between these components and cardiovascular admissions, with black carbon showing the highest impact (1.76% increase per interquartile range), followed by sulfate (1.07%) and ammonium (1.04%). Geographical variations were observed between northern and southern China, with northern cities generally experiencing higher pollutant levels [33].

**Figure 2:**
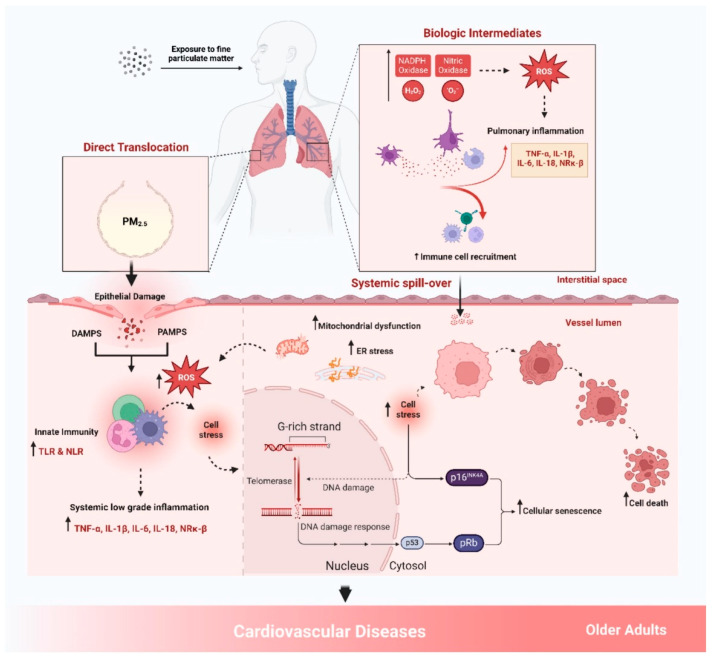
Biological mechanism of PM_2.5_ promoting cardiovascular disease. Reprinted under the terms of the Creative Commons CC-BY license [36].

Key findings from a study revealed that current-day exposure to aluminum (Al) was positively associated with ischemic heart disease death, while cadmium (Cd) and antimony (Sb) showed hazardous effects on CVD mortality at a one-day lag. The effects of heavy metals varied by demographic factors and seasons. For instance, arsenic (As) had a stronger association with CVD death in individuals over 65 years old, and significant associations were observed during summer and winter. Cadmium and lead (Pb) were positively associated with CVD death during summer months. Interestingly, the evidence regarding selenium (Se) was controversial, with some studies suggesting a potential protective effect against CVD [34]. In Lanzhou City, significant associations were found between pollutant levels and CVD hospitalizations. A 10 μg/m^3^ increase in PM_2.5_, SO_2_, and NO_2_ corresponded to relative risks of 1.0013, 1.0040, and 1.0032 for CVD admissions, respectively. Carbon monoxide (CO) showed a particularly strong effect, with a 1 mg/m^3^ increase linked to a relative risk of 1.0909. The impact varied by gender and age. NO_2_ and CO posed greater risks for women, while PM_2.5_ and SO_2_ affected men more significantly. Individuals under 65 were more susceptible to PM_2.5_, NO_2_, and CO, whereas SO_2_ had a greater impact on those over 65 years old. The study also identified lag effects, with pollutants like PM_2.5_, NO_2_, and CO showing the strongest harmful effects at specific lag periods [35]. Collectively, these studies highlight the complex interplay between PM_2.5_ exposure, systemic inflammation, metabolic health, and cardiovascular outcomes. PM_2.5_ exposure leads to the development of metabolic disorders like MetS, which ultimately increase the risk of CVD. Moreover, specific components of PM_2.5_, including heavy metals and organic compounds, may have distinct toxicological profiles that exacerbate cardiovascular risks. Biological mechanisms of PM_2.5_ exposures are presented in Figure 2.

### 2.2. Gaseous Pollutants

#### 2.2.1. Ozone

Ozone (O_3_) exposure has been significantly linked to cardiovascular diseases through multiple biological pathways. It induces respiratory and systemic inflammation, leading to heightened inflammatory responses that exacerbate cardiovascular conditions. Ozone exposure also generates oxidative stress, causing cellular damage and dysfunction within cardiovascular tissues. This oxidative damage impairs endothelial function, disrupts vascular homeostasis, and promotes atherogenesis. Additionally, ozone affects the autonomic nervous system, resulting in alterations in heart rate variability and increased sympathetic nervous activity—factors associated with higher risks of arrhythmias and hypertension. The neuroendocrine system is influenced as well, affecting hormonal regulation critical for cardiovascular health. Moreover, ozone exposure can impair coagulation processes and disrupt glucose and lipid metabolism, further contributing to the development of CVD [37].

Co-pollutant analyses indicated that ozone and NO_2_ might have more pronounced effects on CVD risk compared to PM_2.5_. Similarly, Sun, Gong et al. examined the effects of short-term ozone exposure in Nanjing, China, revealing significant associations between increased ozone levels and cardiovascular mortality [38]. A 10 μg/m^3^ rise in ozone concentration corresponded to a 0.81% increase in cardiovascular deaths (95% CI: 0.49–1.12%) in a multi-pollutant model. Demographic analysis showed greater vulnerability among individuals aged 65 and older, with a trend towards higher risk for females, though this was not statistically significant. From 2013 to 2021, excess deaths attributed to ozone exposure increased alongside rising ozone concentrations. The research estimated that reducing ozone levels to WHO standards could prevent 1736 deaths, while a reduction to minimum levels could save 10,882 lives. Focusing on maternal health, Ruan, Wang et al. revealed a significant association between elevated maternal exposure to ambient ozone during pregnancy and an increased risk of conotruncal heart defects (CTDs) in fetuses [39]. Among 24,278 pregnant women analyzed, 1069 had fetuses diagnosed with CTDs. Exposure to ozone during key pregnancy periods (embryonic, first trimester, and perinatal) was linked to higher CTD risk, with the adjusted odds ratio for each 10 μg/m^3^ increase in ozone during the perinatal period being 1.271 (95% CI: 1.189–1.360). These findings have important implications for public health and maternal–fetal medicine, emphasizing the need for an increased awareness and monitoring of air quality, the implementation of preventive measures to reduce ozone exposure, and guidance for healthcare providers on environmental health assessments.

#### 2.2.2. Carbon Monoxide

Carbon monoxide poses significant risks to cardiovascular health. While it is well-known for its toxicity, it may have therapeutic potential for cardiovascular disease. B-type natriuretic peptide (BNP) and troponin are crucial cardiac biomarkers used in assessing and managing CO poisoning. Troponin, highly specific to myocardial injury, serves as an excellent indicator of immediate cardiac damage caused by CO exposure. Elevated troponin levels strongly correlate with increased short-term risks, such as higher mortality rates and a greater likelihood of requiring intensive care or intubation. This makes troponin particularly useful for guiding immediate treatment decisions and predicting short-term outcomes. BNP provides a broader assessment of cardiac dysfunction, reflecting the degree of heart failure resulting from CO exposure. Higher BNP levels indicate more severe cardiac impairment and are especially valuable for assessing long-term cardiac risks and guiding decisions about treatments like hyperbaric oxygen therapy. The combined use of troponin and BNP enhances diagnostic accuracy and offers a nuanced understanding of both immediate and potential long-term cardiac complications. However, these biomarkers should not be used in isolation; other factors can influence troponin and BNP levels [40]. Research on CO exposure in chick embryos has revealed significant effects on heart development. A key finding was the reduction in heart weight among embryos exposed to moderate CO levels compared to the air control group. Specifically, the average heart weight decreased from 24 mg in the control group to 18 mg in both low- and moderate-CO-exposure groups, with this difference being statistically significant (*p* = 0.013) for the moderate-exposure group. These findings suggest that even low-level CO exposure can negatively impact cardiac development in embryos, which could have implications for human fetal development. This highlights potential risks for pregnant individuals exposed to CO and emphasizes the need for caution regarding air quality and pollution during pregnancy [41].

A positive correlation was observed between ambient CO levels and cardiovascular disease (CVD) hospitalizations. One study demonstrated that a regular increase in CO was linked to a 0.96% higher risk of CVD admissions. Notably, this association persisted even at low CO levels (≤1 ppm), suggesting potential health risks at current ambient concentrations. The researchers employed a comprehensive approach to account for other traffic-related pollutants, utilizing a multisite time-series study design across 126 urban counties over seven years. They adjusted for co-pollutants such as nitrogen dioxide (NO_2_), fine particulate matter (PM_2.5_), and elemental carbon (EC), focusing on same-day pollutant levels. This adjustment attenuated the risk estimates, with the CO-associated risk dropping to 0.55% when accounting for NO_2_ [42]. The heme oxygenase (HO) system, particularly HO-1, plays a crucial role in mitochondrial biogenesis and function by catalyzing the degradation of heme into biliverdin, CO, and iron. CO acts as a signaling molecule with cytoprotective properties, especially under cellular stress conditions. It promotes mitochondrial biogenesis by influencing redox status and affecting transcription factors that regulate mitochondrial growth and function. The HO-1/CO system has been shown to mitigate the progression of degenerative cardiovascular diseases and promote cardiomyocyte maturation. CO’s influence on cardiac function is multifaceted: it regulates cardiac contractility by modulating ion channels, particularly L-type calcium channels; provides cytoprotection against ischemia-reperfusion injury; enhances energy production in cardiomyocytes by promoting mitochondrial function and biogenesis; acts as a vasodilator, improving blood flow to the heart and other tissues; exerts anti-inflammatory effects, protecting cardiac tissue from inflammatory damage; influences cardiac remodeling processes, potentially mitigating adverse remodeling and preserving cardiac structure and function; and affects heart rate and rhythm by modulating autonomic nervous system activity and ion channel function. Given these protective and regulatory roles, CO is being investigated for its therapeutic potential. The pathways through which CO exerts its effects include the mitogen-activated protein kinase (MAPK) pathway, the factor erythroid 2-related factor 2 (Nrf2) pathway, the soluble guanylyl cyclase (sGC) pathway, and the calcium signaling pathways, as well as through mechanisms related to mitochondrial biogenesis and anti-inflammatory effects. These pathways collectively contribute to CO’s role in promoting cell survival, reducing oxidative stress, and maintaining cardiovascular function [43]. Emerging research highlights CO as a potential cardioprotective agent, contrasting with its well-known toxicity. Studies over the past two decades have shown that low doses of CO can protect the heart in models of ischemia-reperfusion injury. Endogenously produced CO is believed to modulate oxidative stress, reduce inflammation, and improve mitochondrial function. Early-phase clinical trials have demonstrated the safety of low-dose CO administration without adverse cardiac events. However, regulatory agencies remain cautious due to CO historical classification as a toxic gas. In cases of CO poisoning, cardiac function is compromised through mechanisms such as hypoxia, increased oxidative stress, impaired mitochondrial function, and altered calcium sensitivity in cardiac myocytes. Acute CO poisoning can lead to arrhythmias, pulmonary edema, and myocardial infarction, while chronic exposure has been associated with cardiac hypertrophy and remodeling. This dichotomy underscores the complexity of CO’s role in cardiovascular health and emphasizes the importance of carefully controlled studies to evaluate its therapeutic potential [44].

#### 2.2.3. Nitrogen Dioxide

Nitrogen dioxide (NO_2_), a prominent air pollutant resulting from vehicular emissions and industrial activities, has been linked to adverse cardiovascular effects. Recent studies have highlighted the significant impact of NO_2_ exposure on cardiac health, particularly its role in exacerbating cardiovascular diseases (CVDs) through various biological pathways. Moreover, ref. [45] conducted a study on patients with dilated cardiomyopathy (DCM) to examine the effects of NO_2_ exposure on cardiac remodeling. The findings revealed that an interquartile range increase in NO_2_ exposure was associated with a 4.3 g/m^2^ increase in indexed left ventricular mass (LVMi) and a 1.5% decrease in left ventricular ejection fraction (LVEF). These adverse effects were more pronounced in women, suggesting a gender-specific vulnerability to NO_2_-induced cardiac changes. The study demonstrated a clear link between higher NO_2_ levels and increased cardiac mass and impaired cardiac function, even after adjusting for confounding factors. Collart, Dubourg et al. explored the relationship between NO_2_ concentrations and hospital admissions for cardiovascular diseases [46]. The research reported an excess relative risk (ERR) of 3.5% for CVD per 10 µg/m^3^ increase in NO_2_. Younger adults aged 25–54 years exhibited a higher ERR of 5.4% for CVD compared to older age groups, indicating a greater susceptibility among the younger population. Gender differences were notable for haemorrhagic stroke, with women showing a higher ERR (7.3%) than men (2.3%). The strongest associations between NO_2_ exposure and hospital admissions were observed on the same day of exposure (lag 0), except for haemorrhagic stroke, which showed the strongest association after a two-day delay. Also, ref. [47] investigated the joint effects of particulate matter (PM_10_) and NO_2_ on emergency cardiac hospitalizations in Hong Kong. The study revealed significant synergistic interactions between these pollutants. When both PM_10_ and NO_2_ concentrations were high, the excess relative risk for all cardiac diseases was 3.75%, while for ischemic heart diseases (IHDs) it was 6.87% and for other heart diseases it was 2.54%. Synergy indices further supported these interactions, with values indicating that the combined effect of PM_10_ and NO_2_ was greater than the sum of their individual effects. On days with high NO_2_ levels (>64.4 μg/m^3^), a 10 μg/m^3^ increase in PM_10_ was associated with a 0.55% increase in cardiac hospitalizations. Conversely, on high-PM_10_ days (>60.9 μg/m^3^), a similar increase in NO_2_ led to a 1.20% increase. These pollutants impact cardiovascular health through mechanisms such as inflammation, oxidative stress, autonomic nervous system dysfunction, enhanced blood coagulability, and vascular dysfunction. Addressing urban–rural disparities, Zhang, Hu et al. analyzed the associations between short-term NO_2_ exposure and increased hospital admissions for various cardiovascular conditions [48]. A 10 μg/m^3^ increase in 24 h NO_2_ exposure was linked to percentage increases in hospital admissions ranging from 1.42% for overall CVD to 2.54% for hypertension on the day of exposure. Urban areas experienced slightly higher percentage increases in CVD hospital admissions (1.51%) compared to rural areas (1.34%). However, rural areas exhibited a more pronounced “displacement” phenomenon, suggesting greater vulnerability among rural populations. The study also emphasized the substantial economic burden associated with NO_2_ exposure, particularly in urban areas where healthcare costs were almost twice as high as in rural areas despite similar increases in hospital admissions.

#### 2.2.4. Sulfur Dioxide

Sulfur dioxide (SO_2_) is a significant air pollutant known to adversely affect cardiovascular health. Recent studies have provided insights into the mechanisms by which SO_2_ exposure contributes to cardiovascular diseases (CVDs), highlighting the importance of addressing SO_2_ pollution for public health. Ref. [49] conducted an extensive study in Guangzhou, China, examining the association between SO_2_ exposure and CVD-related emergency ambulance dispatches (EADs) over the period from October 2013 to June 2018. This association was more pronounced in males, individuals aged 65 and older, and during the cold season. Notably, Guangzhou’s median SO_2_ concentration (11.0 μg/m^3^) was significantly higher than levels observed in Western countries (e.g., 3.1 μg/m^3^ in England and Wales), potentially explaining the stronger associations observed. In a study investigating the molecular mechanisms of SO_2_ impact on cardiac aging, ref. [50] found that the endogenous SO_2_/aspartate aminotransferase 2 (AAT2) pathway is downregulated with aging, leading to SO_2_ deficiency in cardiomyocytes. This deficiency contributes to increased DNA damage and cellular senescence. The mechanism involves SO_2_ modification through sulphenylation at cysteine residue Cys259, which inhibits STAT3 DNA-binding capacity and nuclear translocation. AAT2-knockdown cardiomyocytes exhibited an increased expression of senescence markers (Tp53, p21^Cip/Waf1^, p16^INK4a^) and elevated γ-H2AX foci, indicative of DNA damage. In animal models, cardiomyocyte-specific AAT2 knockout mice showed deteriorated cardiac function and signs of cardiac aging. Importantly, supplementation with SO_2_ donors mitigated these aging phenotypes, suggesting potential therapeutic applications. The study highlights the roles of AAT2 deficiency, DNA damage, cell cycle arrest, and oxidative stress as key contributors to cardiomyocyte senescence.

Mashhadi-Abdolahi, Darbani et al. examined the impact of air pollution, including SO_2_, on cardiovascular health by focusing on oxidative stress and lipid metabolism [51]. Their research indicated that air pollution affects cardiovascular health primarily through inducing oxidative stress, which alters lipid profiles and triggers systemic inflammatory responses. This leads to changes in lipid levels which are associated with cardiovascular events. The study’s data revealed significant differences in oxidative stress markers among exposure groups. Notably, the ambient air pollution (AAP) group showed the highest levels of superoxide dismutase (SOD) and the lowest levels of malondialdehyde (MDA), with statistically significant differences observed between the AAP and control groups for SOD (*p* = 0.05) and between the AAP and ozone groups for MDA (*p* = 0.018). While differences were noted in glutathione peroxidase (GPx) and catalase levels across groups, they were not statistically significant. These studies highlight the significant impact of SO_2_ exposure on cardiovascular health through various mechanisms, including promoting oxidative stress, inflammation, and cellular senescence in cardiomyocytes.

### 2.3. Heavy Metals

Heavy metals are known to be linked to CVDs and heart morbidities.A study by Verzelloni, Urbano et al. found a positive association between cadmium exposure and overall CVD risk, including specific conditions like heart failure, coronary heart disease, and stroke [52]. Importantly, even low levels of exposure showed a linear dose–response relationship, with risk ratios of 2.58 and 2.79 for overall CVD at 1 μg/L in whole blood and 0.5 μg/g creatinine in urine, respectively. Similarly, a study by Rodrigues, Costalat et al. examined the contamination levels of aluminum (Al) and chromium (Cr) in relation to CVD risk [53]. They discovered that 98.6% of participants had Al levels exceeding the allowed limit, with an average concentration of 195.747 µg/L—35 times higher than the reference value. Additionally, 45.8% of individuals had Cr levels above the acceptable limit, averaging 1.524 µg/L, which is triple the acceptable value. Cardiovascular risk assessments indicated that 17.1% of participants were at high risk for CVDs. Notably, those contaminated with both Al and Cr had a 70% probability of high cardiovascular risk, compared to 40% for elderly individuals contaminated with Cr alone. There was a significant correlation between increasing age and higher cardiovascular risk scores, especially in those aged 60 and above. Diet also plays a crucial role in heavy metal exposure. Neisi, Farhadi et al. reported significantly higher concentrations of cadmium and lead in the diets of individuals with CVDs compared to healthy controls [54]. They observed a strong correlation between dietary cadmium intake from vegetables and rice and urinary cadmium excretion, underscoring diet as a major exposure route. Furthermore, the absorption of toxic metals can be influenced by dietary factors, with essential minerals potentially increasing the uptake of harmful metals. Occupational exposure is another critical factor affecting cardiovascular health. Research by Adejumo, Enikuomehin et al. revealed significant cardiovascular risk factors among automobile mechanics compared to control groups [55]. The mechanics showed a higher prevalence of elevated serum cadmium, elevated blood glucose and hyperuricemia. Positive correlations were found between serum cadmium and lead levels with kidney function markers and lipid profiles, indicating increased cardiovascular risk. Additionally, high rates of alcohol consumption (53.1%) and smoking (20.5%) were noted among mechanics. To mitigate these risks, the study recommends implementing health education programs, improving safety measures, enforcing stricter environmental regulations, conducting longitudinal studies, and providing regular health screenings for populations at risk.

### 2.4. Pesticides

Pesticides, widely used in agriculture and households for pest control, have been associated with adverse cardiovascular effects in both humans and aquatic organisms. Recent studies have explored the mechanisms and epidemiological associations between pesticide exposure and cardiovascular diseases (CVDs). A study by Saputra, Lai et al. investigated the cardiovascular effects of fenpropathrin, a type of pyrethroid pesticide, on zebrafish [56]. Exposure to fenpropathrin resulted in significant alterations in cardiac morphology and function. Key findings included cardiomegaly (increased ventricle size) and enhanced cardiac function. Additionally, fenpropathrin exposure led to larger blood-vessel diameters and increased blood-flow velocity. Mechanistically, the pesticide disrupted voltage-gated ion channels, elevated reactive oxygen species (ROS) levels indicating increased oxidative stress and altered the expression of apoptosis-related proteins and cardiovascular genes. These findings suggest that fenpropathrin can substantially alter cardiac performance and morphology, potentially contributing to cardiovascular diseases in aquatic organisms. In humans, Zhao, Li et al. examined the relationship between household pesticide exposure and CVD mortality among older adults using data from the National Health and Nutrition Examination Survey (NHANES) [57]. The study found that exposure to household pesticides was associated with a significantly increased risk of CVD mortality, with an age-adjusted hazard ratio of 1.47 (95% CI: 1.14 to 1.89). This risk was particularly pronounced among older adults with poor diet quality, as indicated by a low Healthy Eating Index (HEI) score. Furthermore, the research identified that elevated levels of urinary metabolites related to household pesticides, specifically DEET from insect repellents, mediated approximately 4.21% of the total association between pesticide exposure and CVD mortality. These findings highlight the potential long-term health effects of household pesticide use and support the link between self-reported pesticide exposure and higher CVD mortality risks.

Moreover, a systematic review by Mohammadkhani, Shahrzad et al. focused on organochlorine pesticides, synthetic chemicals containing carbon and chlorine atoms primarily used in agriculture [58]. Due to their environmental persistence and tendency to bioaccumulate, these pesticides pose significant health risks to humans. The review found a strong association between exposure to organochlorine pesticides and an increased risk of CVD, particularly with early-life exposure. The pesticides exert their effects through multiple mechanisms, including the disruption of metabolic and inflammatory pathways, alterations in peroxisome proliferator-activated receptor gamma (PPARγ) function, the reduction of paraoxonase 1 (PON1) activity, and the induction of oxidative stress in vascular endothelial cells. They also contribute to CVD risk factors such as increased blood pressure, obesity, and type 2 diabetes. Additionally, organochlorine pesticides are linked to endocrine disruption, neurological effects, and potential cancer risks.

### 2.5. Plastics and Plastic Additives

The increasing prevalence of micro- and nanoplastics (MNPs) in the environment has raised significant concerns regarding their potential impact on cardiovascular health. Research has detected MNPs in human blood and tissues, with accumulation particularly observed in areas of vascular lesions. Studies have linked the presence of MNPs in carotid plaques to an increased incidence of serious cardiovascular events, including myocardial infarction, stroke, and all-cause mortality. Experimental data suggest that MNPs can induce oxidative stress, promote platelet aggregation, and trigger inflammatory responses in endothelial and immune cells. These findings underscore the urgent need for large-scale, prospective studies to better understand exposure pathways, establish safety limits, and elucidate the relationship between MNP accumulation and cardiovascular disease development. However, the lack of standardized measurement tools for MNP exposure complicates accurate quantification and risk assessment [59].

Nanoplastics (NPs), in particular, pose significant risks to cardiovascular health through various mechanisms such as oxidative stress, inflammation, and apoptosis in vascular cells. Studies have shown that NPs can infiltrate the cardiovascular system via multiple exposure routes, including ingestion, inhalation, dermal contact, and cellular internalization, leading to structural abnormalities and functional impairments. Both animal and clinical studies have linked NP exposure to altered heart rates, myocardial fibrosis, and increased risks of adverse cardiovascular outcomes like myocardial infarction and stroke [60].

Yalameha, Rezabakhsh et al. examined the effects of plastic particles (PPs) on the cardiovascular system, highlighting their toxicological impacts and mechanisms of action [61]. The study found that PPs can induce oxidative stress, disrupt calcium pathways, and reduce cell viability in heart cells. The toxicity of PPs is influenced by their size and surface properties, with smaller particles (e.g., 100 nm polystyrene nanoparticles) potentially being more harmful. Specific outcomes of the study included arrhythmic phenotypes in heart cells and excessive autophagic responses in endothelial cells. Various types of PPs were studied, including polystyrene nanoparticles (PS NPs) and heavy metal-coated particles, detailing their effects on different cell types. For instance, 50 nm PS NPs induced arrhythmic phenotypes in human embryonic stem cell-derived cardiomyocytes, while 100/500 nm PS NPs caused abnormal autophagic responses in human umbilical vein endothelial cells. A systematic review by Sulistomo, Aditya et al. on micro- and nanoplastic exposure and cardiovascular health revealed significant concerns about their impact [62]. The review examined various plastic types including polyethylene, polypropylene, polystyrene, polyvinyl chloride, and polyethylene terephthalate, with sizes ranging from less than 5 mm (microplastics) to less than 0.1 μm (nanoplastics). Key findings from 38 in vivo studies across different animal models demonstrated that exposure to these particles can lead to cardiotoxicity through mechanisms such as oxidative stress, inflammation, and structural abnormalities in cardiovascular tissues. The severity of cardiovascular effects was found to correlate with particle size, exposure duration, and dosage.

Li, Zhu et al. examined of polystyrene-microplastic (PS MP) exposure effects on heart health in rats, revealing significant adverse impacts [63]. Key findings included increased levels of cardiac injury biomarkers (Troponin I and CK-MB) in serum, particularly in the 50 mg/L PS-MP exposure group. Oxidative stress was evident through elevated malondialdehyde (MDA) levels and decreased antioxidant enzyme activities. Histopathological assessments showed structural damage to the myocardium, including interstitial hyperplasia and vascular congestion. Collagen staining indicated increased cardiac fibrosis, with a higher integrated optical density of collagen in exposed groups. The study also found activation of the Wnt/β-catenin signaling pathway, as evidenced by an increased expression of Wnt, β-catenin, and phosphorylated β-catenin proteins. Additionally, immunohistochemical staining revealed decreased anti-apoptotic signals (Bcl-2) and increased pro-apoptotic signals (Bax), suggesting that PS MPs induce cardiomyocyte apoptosis. These findings collectively demonstrate that exposure to PS MPs can lead to significant cardiac damage, fibrosis, and dysfunction through oxidative stress and the activation of specific cellular pathways. Similarly, Liu, Zeng et al. investigated the effects of polystyrene nanoplastics (PS NPs) on zebrafish, revealing significant cardiotoxic impacts [64]. High concentrations on zebrafish embryos disrupted cardiac development, causing heart malformations and pericardial edema. Continuous 7-day exposure decreased hatching and survival rates. Gene expression analysis showed an upregulation of heart development-related genes (nkx2.5, cmlc-2, myh-7) and Notch signaling pathway genes while suppressing the Wnt signaling pathway gene (wnt-3a). PS NPs induced oxidative stress, activated endoplasmic reticulum (ER) stress, and inhibited mitochondrial activity, leading to cardiac developmental abnormalities. Behaviorally, exposed larvae exhibited increased swimming speed, potentially compensating for impaired cardiac function. Further highlighting the developmental impact, Zhang, Wang et al. investigated the effects of polystyrene microplastics (PS MPs) on myocardial development in birds and chicks [65]. The research revealed significant structural damage to heart tissue, including loose myocardial arrangements and broken fiber bundles. The study found that these effects were primarily mediated through the ER stress-related autophagic pathway, as evidenced by the normalizing effect of 4-phenylbutyric acid (4PBA), an ER stress inhibitor. Statistical analysis confirmed the significance of these findings. The study concludes that PS MPs can induce myocardial dysplasia in birds through ER stress and impaired autophagy, highlighting potential health risks associated with microplastic pollution in ecosystems.

## 3. Clinical Approaches for Monitoring Cardiac Diseases Caused by Environmental Pollutants

**Figure 3:**
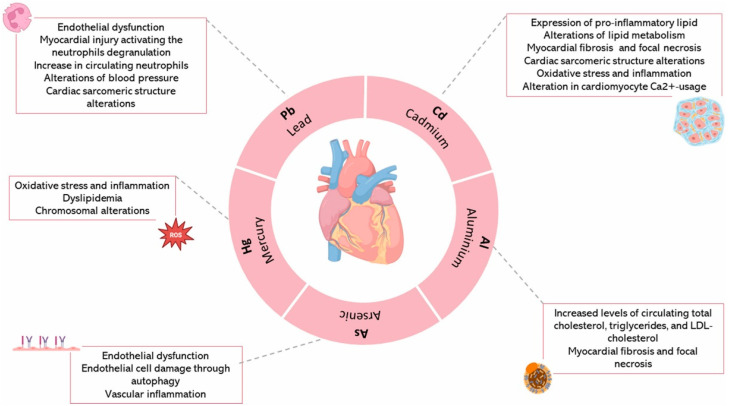
Metabolic and morphological changes associated with cardiovascular system. Reprinted with permission [68].

As cardiac diseases associated with exposure to environmental pollutants have emerged as one of the most pressing health challenges in the last few decades, developing proper clinical approaches for monitoring cardiac diseases caused by environmental pollutants has also gained momentum [66]. Heart attacks and different types of heart failures leading to major cases of mortality and morbidity globally are associated with pollutant exposure [67]. Traditional cardiac risk assessment majorly depends on observing impacts of possible risk factors on the selected population, then leaving provisions for interventions by the scientific community. Figure 3 presents the metabolic changes associated with cardiovascular disease.

### 3.1. Identifying Susceptible Groups

Identifying susceptible groups from a population exposed to environmental pollutants is the first step in clinical monitoring; this assists with identifying vulnerable populations who are at greater risk of developing cardiac diseases when exposed to the same level of pollutants as the rest of the population [69]. As there is no universally accepted data to clearly identify a susceptible group’s possibility of developing cardiac issues related to pollutant exposure, factors like age, gender, socioeconomic status and lifestyle disorders are traced as important markers for scrutinizing the susceptible groups in monitoring studies [70]. The reduced cardiovascular resilience upon aging due to the presence of elastic blood vessels makes older individuals prone to hypertension and associated cardiac issues. Secondly, due to progress in aging, the reduction in immune-cell activity makes it harder to compete with the oxidative damage and inflammations caused by pollutants, which further accelerates the risk of cardiovascular diseases [71]. Furthermore, pre-existing lifestyle disorders like diabetes, obesity, hypertension and other neural issues further exacerbate susceptibility to cardiac issues in risk groups [2]. Several research studies have claimed that children, adolescents, and pregnant women are also at significant risk of developing cardiac disease associated with pollution exposure [72]. Socioeconomic factors also are traced as one of the links that determines the extent of risk of cardiovascular diseases caused by pollutants. For example, the absence of strict legal regulations related to treatment of environmental pollutants in developing countries, along with the high population density living near industrial areas, could also be risk factors that accelerate susceptibility to cardiac issues. Less access to preventive and healthcare services in these countries also elevates this risk [73].

### 3.2. Risk Assessment

Clinical risk assessment to monitor cardiac disease involves routinely used quantitative procedures like blood pressure monitoring, the measurement of cholesterol levels, and imaging techniques. Nevertheless, qualitative risk analysis also plays an important role in monitoring the onset and progression of cardiac issues associated with pollutant exposure [74]. The chief features of various models used for monitoring the impact of pollution exposure on cardiac health are presented in Table 1.

Qualitative procedures—like structured clinical interviews to understand the medical and family history of the patient and analyses of symptoms and risk perception—can provide valuable data about the existing medical conditions of the sampling population and often offer clues related to the possibility of developing cardiac issues in the future. Assessments of the lifestyle and behavior of the sampling population play a pivotal role in understanding the risk factors triggering cardiac issues. This includes dietary habits and factors like alcohol usage/smoking habits, as well as their frequency [81]. The frequency of physical activity (like exercise) and other activities, along with constraints in terms of time/space limitation, other engagements and the medical conditions of the population, also helps in analyzing the possible risk of developing cardiac issues [82]. Risk assessment also involves understanding the psychological and psychosocial factors triggering the development of cardiac issues in a population exposed to pollutants. This includes stress and anxiety due to work or other personal pressure, poor interpersonal relationships and individual social behavior that can accelerate the susceptibility to cardiac diseases [83]. Lastly, several literature sources discuss the impact of environmental and cultural factors on the development of cardiac issues in humans. Cultural and traditional routines involving high salt/sugar-containing food and more reliance on saturated fats like ghee can be traced as risk factors triggering cardiac issues and increasing complications in a population exposed to pollutants [84]. The living environment, especially industrial areas where people are exposed to more air pollutants, poses a greater risk of the development of cardiac issues. Occupational exposure to pollutants related to the nature of work of an individual or a group of individuals (like in agriculture, power plants, and chemical and other industries) could be another factor contributing to the risk of cardiovascular diseases [85].

Quantitative methods for risk assessment may use strategies like personal monitoring, using wearable monitoring devices, and the broader approach of using an exposure modeling system to understand the risk of pollution. Emission models that predict the possible risk based on a particular geographical area are widely used, especially to trace the risk associated with air pollution in causing cardiac issues [86]. Quantitative data from photochemical modelling and meteorological data like the Air Quality Index (AQI) can be used to reflect the possible cases of cardiac issues in a particular geographical area. Another method for quantitative risk assessment involves the use of geospatial risk maps, which rely on models to define short-term cardiovascular risk and long-term cardiovascular risk [87].

### 3.3. Interventions and Recommendations

A major problem that clinicians face during clinical monitoring for cardiac issues is when to clearly decide to intervene and which population should be selected to receive the interventions. There exists a huge diversity in terms of age, gender, sociocultural status, and exposure range among the population exposed to various types of pollutants. Furthermore, differences in the policies and regulations followed by various economies and access to health facilities of the exposed population also create issues in suggesting recommendations during the clinical monitoring of pollution-induced cardiac issues [66]. As the risk-assessment pattern is primarily drawn from epidemiological and toxicological data, there can be many factors that influence the reliability of these interventions. Lastly, the lack of public health policy and regulations and standard protocols for monitoring the impact of pollution exposure on exposed populations also creates difficulties for providing recommendations and conclusions [67].

In addition, community and cohort studies would add new and relevant information on the status of the cardiovascular disease in communities. Cohort studies are major tools used to understand the cause and effect of cardiovascular diseases. Natural products from plants with active ingredients will be useful for the development of drugs to cure cardiovascular disease. Moreover, nanomedicine will help in the development of new products to combat the problems associated with cardiac health.

## 4. Conclusions, Challenges and Future Directions

The current review discusses the sources and negative impacts of environmental pollution on cardiac health. Emerging environmental pollutants and their corresponding cardio-destructive roles have been promptly investigated and documented [88]. Clinical diagnosis and various approaches available so far to identify, monitor, and cure cardiovascular diseases caused by environmental pollutants have been briefed with probable interventions [89]. Thus, environmental pollutants causing cardiovascular diseases were unfortunately unknown for a considerable time, and they are still overlooked. Such environmental risk factors trigger cardiovascular stress, intensifying it into cardiovascular diseases that put life at risk [90]. Lifestyle patterns could certainly restrict human exposure to such environmental pollutants, but their complete eradication is inevitable due to their ubiquitous nature. Choices over lifestyle will not aid sufficiently to avoid cardiac health risks due to environmental pollution. Conventional waste management practices such as landfills, dumping and incineration are proven to be problematic in terms of inhabitants’ risk and environmental contamination [91]. The accumulation of pollutants (plastic-derived materials, particulate matter, heavy metals and gaseous contaminants) and polluted water and air are an unpreventable end result of the rapid expansion of cities across the world [92] air. Resource overconsumption stays never-ending, deteriorating the environment and society. Finding new ways of reducing cardiovascular problems due to environmental pollutants is a topic that must be a priority for every government, business and individual [93]. People’s lives should be marked with instantaneous cardiac risk-assessment strategies due to environmental pollution, medical interventions (treatments), lifestyle recommendations and advice regarding decreasing exposure, allowing for viable environmental practices. Uncompromising legislation to prevent environmental pollution and life-threatening pollutants should be effectively enforced by the ruling governmental bodies [94].

Miserably, environmental pollutants are yet to be specified as emerging risk factors for cardiovascular diseases and are to be included in the American College of Cardiology (ACC) and American Heart Association (AHA) guidelines, lacking much-needed attention. Information about the effects of various environmental stressors on the human cardiovascular system is perhaps scarce in the European Society of Cardiology (ESC) guidelines of clinical practice [95]. The challenge lies in researching the negative additive effects of environmental pollutants, in vitro and in vivo, on the cardiovascular system. The literature shows an absence of appropriate animal modeling and human research. Each environmental risk factor and its molecular mechanism (pathway) affecting the human cardiac system has to be thoroughly studied [96]. Though the fundamentals of the environmental pollutants’ cardiovascular effects have been sketched well—including their individual (independent) and co-exposure (interactive) effects and the impact of time period, intensity, transformational ability, breathing space and circadian rhythm alterations/rejuvenations—advanced and comprehensive findings on preventive measures and medical care are necessary [97]. The interactional effects of environmental pollutants with biological (non-environmental) risk factors and their irreversible effects on human health remain the biggest question to be answered. However, high research costs and response burdens faced during such extensive studies hamper technological development and data validation [98].

Regardless of notable increases in the informational data and knowledge obtained during the last several years, a handful of significant questions remain unanswered [99]. More cutting-edge technologies and research studies are essential to understand the complete elucidation of dose–response assessments of environmental pollutants. This has a massive consequence for human cardiac health globally [100]. As the human exposure to such environmental pollutants increases, risks associated with cardiovascular diseases also increase, enabling supralunar response [101]. The socioeconomic status and age of individuals are the most challenging factors that provoke the negative consequences of environmental pollutants. In order to fix the standards and guidelines, a greater comprehension of the vulnerable and susceptible human groups will ultimately aid in saving the public’s health [102,103]. Out of the numerous environmental pollutants, challenges lie in identifying the most unsafe and virulent environmental stressors and their corresponding sources, for which distinct short- as well as long-term analyses with statistical interpretations have to be designed and exercised [104]. Problems associated with handling cardiovascular issues due to multiple environmental pollutants, their accuracies, wide-scale measurement facilities, population clustering, diagnosis, and treatments with large-scale inter-correlations and complicated study designs are to be resolved [105]. Identification methods and statistics indulged during cluster studies have to be carefully chosen for a better understanding of the affected population [106]. The mechanism and complexity of each environmental pollutant differ, making it hard to streamline and requiring extensive research. Regulatory policies about the harmful effects of environmental pollutants on the cardiovascular system should be solidly built; this could lead to higher-level preventive outcomes [107].

Globally, myocardial infarctions and traffic exposure on a daily basis are interconnected; traffic pollution plays a significant triggering role in injuring the cardiac functions. Among the various environmental pollutants, air pollutants rank first for their harmful and devastating effects on cardiac health [108]. It is very difficult to distinguish gaseous particles from other forms of environmental pollutants (for instance, temperature and noise), and air pollutants’ ill effects are highly detrimental and laborious to recognize individually [109]. The lack of an absolute understanding of multiple exposures to combinations of traffic-related microparticles, noise pollution and gaseous air pollutants—as causative agents of human cardiovascular diseases and psychological problems, especially for communities and populations near highways—needs to be quickly addressed, considering both individual and synergistic negative effects [110]. Public (population) health studies have strongly evidenced many incidences of cardiometabolic malfunctions in people who live near highways and contaminated areas, which need immediate attention [111]. High-end research works investigating traffic allergens and pollutants are mandatory, specifically regarding cardiovascular diseases [112].

An IEC, or Information, Education and Communication, campaign is a method wherein reaching the basic or last member of the community is feasible. IEC campaigns on possible preventive measures and the minimal exposure of vulnerable populations near roadways and polluted zones to environmental pollutants are appreciated [5]. An IEC campaign can also be achieved by using the educational sector (schools, colleges and research centers) as a means to educate students regarding the emerging environmental concerns for cardiac diseases, so that the values of cleanliness and hygiene amongst children can be instilled in a bid to build the next generation of waste-conscious citizens. Tarpaulins and flyers as a way of reaching people can inculcate practices that could create cardiac health awareness and also reduce exposure to environmental pollutants [113]. Using visual media is a very good approach to informing, educating and communicating the community, especially children, to create ambassadors (key influencers) of change who transmit messages in their communities of reach. This will inform people about the ‘dos’ and ‘don’ts’ of human exposure to pollutants, educate people about the importance of eco-friendly environmental practices, and facilitate communication between the community and the governing body, which will then take over and establish reinforced regulatory policy systems [114].

Despite the fact that environmental pollutants have demonstrable destructive impacts on human cardiovascular health, there remains an enormous range of solutions to alleviate direct exposures and mitigate their ill effects [5]. Further interventions must be carried out at individual, decentralized, centralized and/or even universal levels. Regulatory policies and structural modifications, in order to reduce environmental pollution and thereby pollutants, may eventually contribute to decreased cardiac issues [115]. Urbanization with greenness is necessary to build healthy cities. Examples include domestic alterations to reshape the existing urban environment through using eco-friendly materials to construct buildings, establishing green spaces, and executing building design and planning in such a way that the density of the residential population and their transport infrastructure is carefully kept in mind and features advanced noise barriers [116]. Health risk assessment is also an important parameter to minimize cardiovascular disease risks [117]. This can remarkably reduce cardiovascular diseases due to environmental pollutants. Transitions are expected in the prevailing transportation systems and legislations [118]. Attempts to move to electric vehicles from vehicles made of combustion engines, vehicle speed control/barrier systems, noise control and the development of green automobile systems with low gas emissions and noise are warranted to alleviate environmental pollutants causing cardiovascular problems [119].

An increase in naturalness (greenness) is directly associated with a decrease in mortality rates due to cardiovascular disease. This statement needs more authentication in terms of research and the development of green solutions to reduce the generation of and exposure to environmental pollutants [120]. Apart from such time-consuming centralized regulations that need high societal awareness and interventions by local governing bodies, individual-based transitions in lifestyle could support healthy living, with minimal exposure to environmental pollutants [121]. Personalized and portable air purifiers (cleaners), respirators, masks and related protection devices may aid in the reducing exposure to environmental pollutants, lessening the probability of developing cardiovascular diseases [122]. Green foods and purified water can reduce exposure to heavy metals and other emerging environmental pollutants [123]. Lifestyle and behavioral effects can profoundly influence the human cardiovascular system, even in a contaminated environment. Preventive measures and actions—for instance, physical exercise and healthy living in safer environments—are advisable [124]. Outdoor physical activities, especially during old age, will safeguard cardiovascular health. In whatever way, a single action of humans will not reduce the cardiovascular effects of the existing environmental pollutants. In this case, the preventome needs multifarious research, interventions and effective legislations in local as well as regional levels, over time, just like its corresponding exposome [125]. Hence, the future cardiovascular disease burden due to environmental pollutants could probably be hindered by careful personal care and monitoring, efficient mitigation attempts, green urban planning and governmental interventions. Although sophisticated medical techniques and treatments are readily available for cardiovascular diseases worldwide, management efforts to reduce emerging risk factors like environmental pollutants must be implemented to their fullest, instead of fully focusing on traditional risk factors like lifestyle, genetics and biochemistry. Day by day, strong evidence emerges proving that environmental pollutants (also known as stressors, risk factors and toxins) are influential factors for human cardiovascular risks. Environmental pollutants as emerging contributors to cardiac diseases serve as a wide essential platform for researchers and medical doctors to reduce environmental pollution, which is reversible (modifiable). This study highlights the growing impact of environmental pollution on heart health, which is often overlooked as a major cause of cardiovascular diseases (CVDs). Micropollutants, harmful gases, and microplastics worsen heart disease, with effects that vary by region and vulnerable population. The evidence shows that chronic exposure to these environmental risks, especially in cities, increases the chances of myocardial ischemia, high blood pressure, stroke, and death from heart-related problems. Therefore, it is clear that addressing environmental pollution should be a part of the efforts to prevent and manage heart disease. Public health strategies, such as stricter pollution regulations, urban planning that includes more green spaces, and educational campaigns, are important to reduce exposure to these pollutants. Furthermore, new tools like wearable devices, exposure modeling, and geospatial risk assessments can help in identifying people at higher risk. By also considering factors like socioeconomic status, lifestyle, and work conditions, we can better understand which individuals are most at risk of pollution-related heart damage. This study calls for a team-based approach to deal with the risks pollution poses to heart health. Strengthening laws, working together across healthcare, environmental science, and policy, and developing focused interventions will be key to reducing pollution’s negative effects on heart health. As more research is conducted, it is important to better understand the long-term impact of new pollutants like microplastics and improve risk-assessment methods for better public health strategies. In summary, addressing environmental factors that contribute to heart disease is crucial for reducing cardiovascular diseases, improving public health, and creating healthier environments for future generations.

One of the challenges in interpreting observational studies on environmental pollution and cardiovascular diseases is the potential for reverse causation and confounding biases. Reverse causation refers to a situation where the outcome (e.g., cardiovascular disease) may influence the exposure (e.g., pollution levels) rather than the exposure causing the outcome. For instance, individuals with pre-existing cardiovascular conditions may reside in areas with higher pollution due to socioeconomic factors, leading to biased associations. To address this, studies included in this review often employed advanced statistical methods, such as propensity score matching, instrumental variable analysis, and multivariable regression, to minimize biases. For example, stratified analyses based on demographic variables, adjustment for co-pollutant exposures, and the inclusion of longitudinal designs have been used to strengthen causal inferences. While these approaches improve reliability, the inherent limitations of observational data necessitate caution in drawing definitive conclusions. Future research should aim for more robust designs, such as randomized controlled trials or natural experiments, to establish clearer causal pathways between pollution exposure and cardiovascular outcomes.

## Figures and Tables

**Table 1:** Different models for assessing the impact of pollution on the cardiac health of the exposed population.

Model Type	Significant Feature	Advantage	Disadvantage	Reference
Proximity based methods	Relies on the location between individuals/populations location from the source of pollution	Simple method, Special training and expertise not required	Comparative studies between different data derived from various geographical locations are difficult	[75]
Statistical methods	Employs geostatistical techniques	Simple in operation	Variability in data between various locations may affect the data interpretation process	[76]
Land use regression models	Uses multiple predictors to derive data about exposed population	Provides spatially resolved predictions	Suitable only for long time exposure studies	[77]
Dispersion Models	Depends on Gaussian plume equations	Takes in to account both partial and temporal variation in pollution	Needs costly data input, unrealistic assumptions and possibility of estimate errors	[78]
Chemical transport models/Integrated Meteorological-Emission Models	Involves combination of chemical methods and meteorological data	Highly reliable computational method	Computationally intensive procedure requiring extensive data input and specific expertise for data collection and interpretation	[79]
Hybrid models	Employs combination of two or more models to derive resolved data	Greater accuracy and reliability	Method requires time, computational support and skilled person for data collection and validation	[80]
